# The Spectrum of COVID-19-Induced Liver Injury in Various Age and Risk Groups

**DOI:** 10.7759/cureus.36349

**Published:** 2023-03-19

**Authors:** Tapasya Bhusal, Prakash Banjade, Salim Surani, Munish Sharma

**Affiliations:** 1 General Medicine, Shahid Dharmabhakta National Transplant Center, Kathmandu, NPL; 2 General Medicine, Manipal College of Medical Sciences, Pokhara, NPL; 3 Medicine, Texas A&M University, College Station, USA; 4 Medicine, University of North Texas, Dallas, USA; 5 Internal Medicine, Pulmonary Associates, Corpus Christi, USA; 6 Clinical Medicine, University of Houston, Houston, USA; 7 Pulmonary and Critical Care, Baylor Scott & White Medical Center - Temple, Temple, USA

**Keywords:** alanine transaminase, aspartate transaminase, liver enzyme, liver disease, sars-cov-2 infection liver injury in covid-19, coronavirus disease-2019

## Abstract

Coronavirus disease 2019 (COVID-19) has inflicted significant mortality and morbidity worldwide since the virus was first detected towards the end of 2019. Though it primarily affects the respiratory system, COVID-19 has been shown to have a multisystem effect. There have been literature on liver injury associated with COVID-19 in general but liver injury specific to certain risk and age groups needs to be looked into. Thus, we aim to discuss the liver injury associated with COVID-19 in various age and risk groups and revisit pathophysiology, biochemical markers and their correlation with outcomes, and current management recommendations.

## Introduction and background

Coronavirus disease 2019 (COVID-19) is a viral infection caused by the SARS-CoV-2 virus [1]. Ever since its emergence, it has been a public health challenge. On March 11th, 2020, World Health Organization (WHO) declared the coronavirus disease 2019 (COVID-19) a global pandemic. COVID-19 has caused around 6,866,434 deaths globally [1]. COVID-19 affects various organ systems in the body, and several studies have shown the effects of the virus on the hepatobiliary system. The aim of this review article is to review the existing literature on COVID-19-induced liver injury with special emphasis on its effect among various age and risk groups.

## Review

Pathophysiology of liver injury in COVID-19

Located between the portal and systemic circulations and continuously being exposed to dietary antigens, viruses, and other inflammatory products, the liver is at the highest risk of getting damaged by exposure to toxins, bile duct obstruction, viral infections, and preexisting chronic liver conditions [2]. The exact underlying cause of liver damage in COVID-19 and its treatment course is still not clearly established. However, various mechanisms have been suggested as discussed subsequently.

Cytopathogenic Effects of the Virus on the Liver Cells

In COVID-19, liver injury might be the virus's direct effect on the liver cells. Chai et al. [2] performed a study on four donors who had sustained cardiac arrest. They performed sequencing of RNA from the liver tissue samples of these four donors. They found that both hepatocytes and bile duct cells expressed ACE2 receptors that had a high affinity for the spike protein of SARS-CoV-2. There was a higher expression of ACE2 receptors in cholangiocytes. This could be compared to the ACE2 receptor expression in type II alveolar cells, which is generally almost 20 times higher than the liver cells [2]. Analysis of two post-mortem liver biopsies of patients infected with COVID-19 showed apoptotic hepatocytes with moderate microvesicular and mild macrovesicular steatosis portal and periportal inflammation. Obvious viral inclusion was not noted. Immunohistology report revealed numerous CD68+ cells in sinusoids and scattered CD4+ and CD8+ cells in lobules [3]. These findings are suggestive of immune-mediated hepatocyte damage rather than a direct effect of the virus on liver cells. Cholangiocytes play a vital role in liver regeneration and immune responses, so the effect of the COVID-19 virus on these cells may lead to profound consequences in the liver [3]. Since viral inclusion was not noted, it is highly likely that cytopathogenic effects of the virus could be a possible mechanism of injury.

Cytokine Storm

Different types of pro-inflammatory cytokines have been found to play a role in the cytokine storm. Initiation of cytokine storm heralded by mediators such as Interleukins 2,6,7 and 11, interferon-gamma, and tumor necrotic factor alpha ultimately leads to multi-organ failure (MOF). It can also cause a hypercoagulable state. An imbalance in coagulation homeostasis as a result of systemic inflammation leads to a condition of a pro-coagulant state manifesting as micro-thrombosis, disseminated intravascular coagulation, and multiorgan failure [4].

Ischemia and Reperfusion Injury

There can be an initial ischemic insult to the hepatocytes in COVID-19. This is considered a localized process comprising disturbances of lipid metabolism, adenosine triphosphate scarcity, and glycogen consumption that collectively leads to cellular death. During the reperfusion phase following the ischemic injury, there is massive recruitment of pro-inflammatory immune cells from the circulation that leads to a reperfusion injury to hepatocytes [4].

Liver Injury in COVID-19 Due to Drugs That Were Once Used or Are Currently Being Used for Treatment Purposes

**Figure 1:**
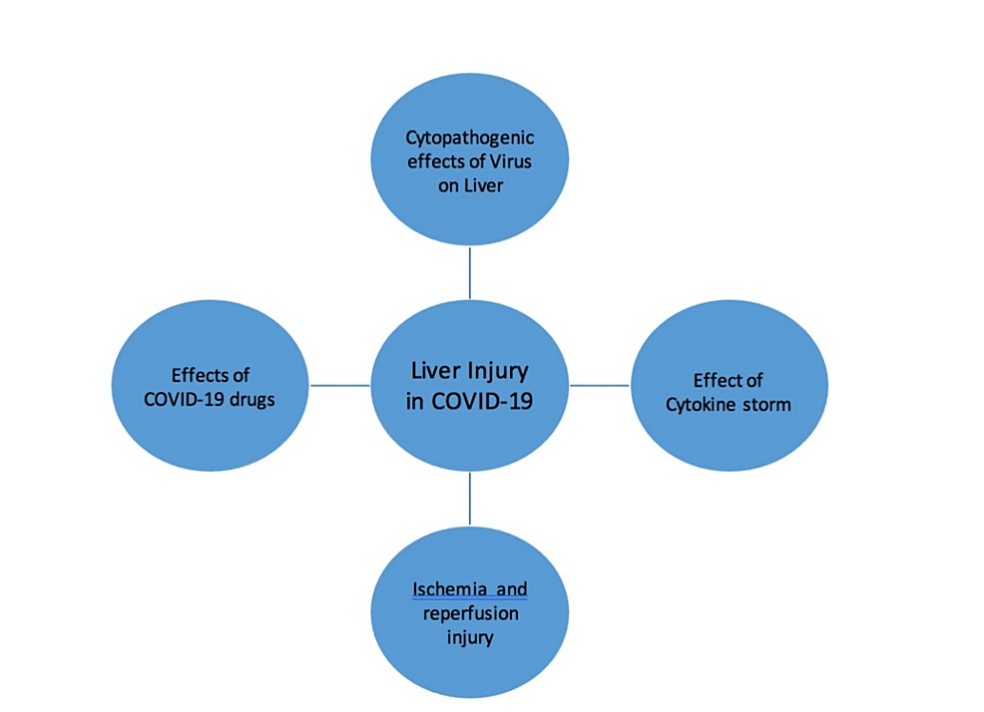
A flow diagram describing pathophysiology of liver injury in coronavirus 2019 disease The figure is created by the authors.

Various drugs like chloroquine, azithromycin, acetaminophen, remdesivir, lopinavir/ritonavir, tocilizumab, and corticosteroids that were proposed to be used for treatment in COVID-19 or that are still in use may be the potential cause of hepatocyte injury in patients with COVID-19. An increase in bilirubin levels, along with alteration in aspartate aminotransferase/alanine transaminase (AST/ALT), was seen with the use of lopinavir/ritonavir in patients with preexisting liver disease [5]. Lopinavir/ritonavir is not recommended anymore. Transient increase in AST/ALT was evident with the use of an anti-IL-6 receptor monoclonal antibody called Tocilizumab. Such transaminitis was found to be generally transient and subsided within a period of around six weeks. Remdesivir is an antiviral agent that was approved for the treatment of patients with severe pneumonia due to COVID-19. In a study by Zampino et al., there were five cases of COVID-19 who were treated with remdesivir. They had elevated AST/ALT levels, suggesting hepatocellular injury without liver failure [6]. Hydroxychloroquine and azithromycin are associated with drug-induced liver injury, as evident in a randomized controlled trial involving 667 patients. These patients received either hydroxychloroquine as a single agent or a combination with azithromycin [7]. It is a well-established fact that antipyretics such as acetaminophen can have a dose-dependent effect on the hepatocytes. Patients with preexisting chronic liver diseases are more vulnerable to the effects of acetaminophen. They can have hepatoxicity even with lower doses of acetaminophen. Since patients with COVID-19 infection can have persistent fever for a certain duration of time, there is a high propensity to have a liver injury from antipyretics agents themselves. The United States Federal Drug Administration (USFDA) even lowered the maximum recommended dosage of acetaminophen to 3 grams in 24 hours, especially in patients at risk of liver injury [4]. Thus, the pathophysiology of liver injury in COVID-19 is complex and can be summarized in a flow diagram, as shown in Figure 1.

Liver injury in various age groups and risk population

Hepatic injury in the pediatric population with COVID-19 generally manifests as mild elevation in AST and ALT without derangement in the synthetic function. Acute liver failure is reported to be uncommon in the pediatric population. However, a few cases of acute liver failure are reported in the literature. Antala et al. reported a case series of four pediatric patients with COVID-19 who did not have any pre-existing liver disease. None of them had pulmonary symptoms due to COVID-19, but two of the four patients had acute liver failure. The authors opined that it resulted from complement activation and reported that one of the children was treated successfully with eculizumab. Rest three children improved just with supportive measures [8]. All four pediatric patients had an elevation in AST, ALT, and total bilirubin. Two of these patients met the criteria of the Pediatric Acute Liver Failure Study Group, PALFSG, for acute liver failure, as reported by Antala et al. [8]. The age group ranged from 4 months to 16 years. The length of stay in the hospital, including the ICU and ward, was 4 days to 18 days. Liver function tests (LFTs) improved at the time of discharge and normalized in their follow-up visits within 1-6 months (Table 1) [8].

**Table 1:** Liver injury in coronavirus disease 2019 in the pediatric population AST: Aspartate aminotransferase, ALT: Alanine transaminase

Study population	Authors	Study origin and date	Study type, sample size	Key findings
Pediatric population	Antala et al. [8]	United States. May 2022	Case series, n=4 patients	Age range: 4 months to 16 years, Sex: Female 2, Race: African American 2, Caucasian 2, Peak ALT range: 1932 to 11,150, Peak AST range: 850 to 10,110, Peak INR range, 0.87 to 7.4, Recovery of Liver enzymes: 1 to 6 months
Pediatric population	Di Giorgio et al. [9]	Italy, Germany, United Kingdom. March 2021	A systematic review of 105 studies, N=369	Mean age: 11.1 +/- 7.7 years, Sex: Male 60% (220), Survival: 369 cases
Pediatric population	Qiu et al. [10]	Zhejiang, China. June 2020	Retrospective review, N=36 patients from 3 hospitals	Mean age: 8.3 +/- 3.5 years, Sex: Male 64% (23), ALT elevation 2 patients, AST elevation 3 patients, Liver failure: None
Pediatric population	Wang et al. [11]	Northern China. April 2020	Retrospective study, N=31 cases	Age range: 6 months to 17 years, AST/ALT elevation in 22% of cases. Peak ALT 68 International units/Liter (IU/L), Peak AST 67 IU/L, Liver failure: None. Total recovery and discharge 77%, 24 children
Pediatric population	Cai et al. [12]	China. September 2020	Retrospective study, N=417 total cases, Pediatric 34	Age < 10 years, 20 patients. 10-19 years, 14 patients. Total patients with elevated AST/ALT 76.3%. No mention of pediatric liver failure

Giorgio et al. performed a systematic review concluded in March of 2021. They included 105 contemporary studies with a total sample size of 369 pediatric population. The authors concluded that children get a milder liver injury as compared to adults. The authors believe that the activated lymphocytes can get rid of the stimulus (viral antigen) more effectively. Children have an intact effector and immunosuppressive capacity as compared to adults. They reported that all 369 pediatric patients survived. Children with chronic liver disease, autoimmune liver disease, and transplantation were all devoid of any major complications even when they were infected with COVID-19. The authors have also requested pediatricians to consider underlying chronic liver disease in children with COVID-19 with deranged liver enzymes, referring to the fact that 1 to 11% of these patients admitted with COVID-19 had underlying chronic liver disease [9]. In a retrospective review of 36 children from three different hospitals performed by Qiu et al., only two pediatric patients with COVID-19 were found to have ALT elevation, while three had AST elevation. None of them had a failure of the synthetic function of the liver. Fourteen of these patients were treated with lopinavir-ritonavir, while 36 of them had received interferon alpha as a part of their COVID-19 treatment [10]. In another retrospective study from Northern China, 22% of pediatric patients admitted with COVID-19 had an elevation in liver enzymes without any evidence of liver failure [11]. In a study of 417 patients with COVID-19, out of which 34 patients were below 19 years of age, the authors reported that a high number of patients had elevated AST/ALT (76.3%). There was no definite distinction for the pediatric population, but no mention of pediatric liver failure was found (Table 1) [12].

The impact of COVID-19 on maternal and neonatal outcomes has not been properly documented. In one study, out of 249 women who tested positive for COVID-19, 42.97% (107) had acute liver function abnormality upon admission, and 57.03% (142) had a normal liver function. Based on the severity of COVID-19, women were categorized as asymptomatic, mild, moderate, and severe COVID-19. Pregnant women with liver injury had severe COVID-19 disease and had deranged LFTs. In the group with liver dysfunction, a greater percentage of pregnant women underwent cesarean section compared to the group with normal liver function; but this difference was not statistically significant. In the group of pregnant women with hepatic dysfunction associated with SARS-CoV-2 infection, postpartum hemorrhage, the requirement for blood transfusion, complications like sepsis and failure of multiple organs, and mortality were more common [13].

In another retrospective cohort study involving 122 pregnant women with COVID-19, 17 (13.95%) patients had abnormal LFTs during hospitalization. Critically ill patients were more in number in the abnormal LFTs group than in the normal LFTs group. In the group of pregnant women with abnormal LFT, the use of medications lopinavir/ritonavir and hydroxychloroquine was observed to be significantly higher, with prolonged treatment duration and hospitalization [14]. These findings imply that close monitoring of liver function tests should be done for pregnant women infected with COVID-19 who received antiviral treatment. Various co-morbid conditions in patients like hypertension (HTN), diabetes, chronic kidney disease (CKD), asthma, chronic obstructive pulmonary disease (COPD), chronic liver disease (advanced fibrosis and cirrhosis), non-alcoholic fatty liver disease (NAFLD) or non-alcoholic steatohepatitis (NASH), hepatitis B virus (HBV), hepatitis C virus (HCV), autoimmune hepatitis, primary biliary cirrhosis (PBC), and hemochromatosis were studied. HTN and diabetes were the most common comorbidities, and both were inversely associated with a higher category of peak ALT elevation (p<0.001). CKD was associated with the higher category of peak ALT elevation (p<0.001) [15].

Liver injury in COVID-19 patients with previous liver disease

Among the patients at risk of getting an acute liver injury in COVID-19, those with pre-existing liver conditions are at risk. We aim to focus on the prevailing evidence about this vulnerable group in this separate sub-topic. The patients with chronic liver diseases such as alcohol-related liver disease (ALD), decompensated cirrhosis, and hepatocellular carcinoma who are infected with COVID-19 have a heightened risk of mortality from COVID-19 as revealed in one study, including 867 patients with chronic liver disease with COVID-19 from 21 centers across the United States [16].

According to the study results, both Hispanic ethnicity and the presence of decompensated cirrhosis were found to be independent risk factors for severe COVID-19 outcomes [17]. Another study included 133 cases with 116 patients hospitalized with COVID-19 with serum HBsAg negative (SARS-CoV-2 group) and 17 HBV inactive carriers with COVID-19 (HBsAg positive, HBeAg-negative with undetectable HBV viral load SARS-CoV-2/HBV co-infection group, clinical indicators associated with liver injury were analyzed for three weeks, which demonstrated that inactive HBV carriers with SARS-CoV-2 are prone to have abnormal liver functions tests; with cellular injury more in hepatocytes compared to cholangiocytes. At the same time, no significant differences were noted in the rate of discharge from the hospital or length of hospital stay among the two groups. The co-infection exacerbates the liver function of patients with COVID-19 [17].

Another study revealed that the higher occurrence of non-alcoholic fatty liver disease (NAFLD) increases the risk of a significant portion of the population experiencing complications of COVID-19 [18]. Patients infected with COVID-19 with a history of liver transplantation recipient surgery have a high chance of acquiring severe pneumonia with hospitalization in the Intensive Care unit requiring mechanical ventilation. In addition, this group of patients was found to have a greater risk of liver injury, as shown by changes in liver function tests. The risk was related to the degree of immunosuppression and the presence of comorbid conditions like diabetes, cardiovascular and renal disease, and an increased risk of medications and their interactions [4]. Liver injury is an independent risk factor for increased mortality. This also signifies the need to monitor LFTs in liver transplant recipients with COVID-19 as a predictor of liver injury and in recognizing patients who are at risk of unfavorable outcomes (Table 2) [19].

**Table 2:** Liver injury in coronavirus disease in pregnant and adult population AST: Aspartate aminotransferase, ALT: Alanine transaminase, COPD: Chronic obstructive pulmonary disease, CKD: Chronic kidney disease, HTN: Hypertension, NAFLD: Non-alcoholic fatty liver disease, NASH: Non-alcoholic steatohepatitis, HBV: hepatitis B virus, HCV: Hepatitis C virus, ULN: Upper limit of normal, TBIL: Total bilirubin.

Study population	Authors	Study origin and date	Study type, sample size	Key findings
Pregnant population	Choudhary et al. [13]	India, May 2022	Retrospective observational cohort study, N=249	Age: Mean age 28.38 years, Mean period of gestation in cases with liver dysfunction: 35.42 weeks, Lab findings consistent with liver dysfunction: 42.97% of cases
Pregnant population	Can et al. [14]	Turkey, October 2021	Retrospective cohort study, N=122	Age: 28.8±6.8 years, Gestational weeks: 29.4±9.3 weeks, Peak AST value: 207 U/L Peak ALT value: 200 U/L
Adult population	Kim et al. [16]	United States, 2021	Multicenter observational cohort study, N=867	Age: Mean age 56.9±14.5 years, Sex: 54.7% male patients, New or worsening hepatic decompensation during COVID-19A: 67 patients (7.7%). Severe hepatic encephalopathy: 23 patients (32.3%)
Adult population	Cai et al. [12]	China, April 2020	Cross-sectional study, N=417	Age: Median age 47 years, Abnormal Liver Function Tests Results: 76.3% of cases. Liver Injury: 21.5% of cases
Adult population	Lei et al. [20]	China, April 2020	Multicenter retrospective cohort study, N=5771	Age: Median age 56 years, Sex: 47.2% male; Comorbidities: COPD, DM Type 2, Hypertension, Coronary heart disease, Cerebrovascular disease, Chronic liver disease, CKD, Cancer, Autoimmune disease Elevated AST between 40 U/L and 120 U/L: Increased risk of all-cause mortality by 4.81-fold; 28-day mortality estimation adjusted HR 6.00. Elevated AST above 120 U/L: Increased risk of mortality by 14.87-fold; 28-day mortality estimation HR 17.05
Adult population	Guan et al. [21]	China, February 2020	Cross-sectional study, N=1099	Age: Median age 47, Sex: 41.9% female patients; Co-morbidities: COPD, Diabetes, Hypertension, Coronary Heart disease, Cerebrovascular disease, Hepatitis B infection, Cancer, Chronic Renal Disease, Immunodeficiency Raised AST >40 U/L: 22.2%, Raised ALT >40 U/L: 21.3%, Raised Total Bilirubin >17.1 µmol/L: 10.5%
Adult population	Phipps et al. [15]	China, May 2020	Retrospective cohort study, N=2273	Age: Median 65 years, Sex: 57% male; Comorbidities: HTN, Diabetes, CKD, Asthma, COPD, Chronic liver disease (advanced fibrosis and cirrhosis, alcohol-related liver disease, NAFLD or NASH, HBV, HCV, Autoimmune hepatitis, Peak ALT/Initial ALT: >ULN 45%/24%; >5×ULN 6.4%/1.3%, Peak AST/Initial AST: >ULN 74%/56%; >5× ULN 13%/3.8%
Adult population	Hundt et al. [22]	United States, October 2020	Retrospective cohort study, N=1827	Age: Mean age 65 years, Sex: 53% male, Comorbidities: Obesity, diabetes mellitus. Abnormal Liver Tests during peak hospitalization/Pre-hospitalization AST: 83.4%/20.3%, ALT: 61.6%/19.1%, ALP: 22.7%/13.4%, TBIL: 16.1%/4.1%, AST >5×ULN: 16.6%, ALT >5×ULN: 20.6%

Biochemical markers of liver injury in COVID-19 and data on their correlation to outcomes

The degree of liver injury is measured in terms of changes in liver enzymes; level of ALT >40 U/L, AST >40 U/L, gamma-glutamyl transferase (GGT) >49 U/L, alkaline phosphatase (ALP) >135 U/L, and total bilirubin (TBIL) >17.1 micromol/L were defined as abnormal liver test results [8]. Furthermore, an elevation in ALT and/or AST over three times the upper limit of normal (ULN) and an increase in ALP, GGT, and/or TBIL over two times ULN is defined as liver injury [12]. In one cross-sectional study done in China, among 417 patients diagnosed with COVID-19, 318 (76.3%) had abnormal test results, and 90 (21.5%) had a liver injury during hospitalization [20]. Another study conducted in China, involving 5771 adult patients with COVID-19, evaluated the correlation between increased levels of ALT, AST, ALP, and TBIL with all-cause mortality. The risk of all-cause mortality was significantly increased 4.81-fold (95% CI, 3.38-6.86; P<0.001) in patients with AST between 40 U/L and 120 U/L compared to patients with AST in the normal range and increased 14.87-fold (95% CI, 9.64-22.93; P<0.001) in patients with AST above 120 U/L taking into account factors such as age, gender, and pre-existing health conditions. In non-severe patients, liver injury was mild and short-term. The increase in AST levels was more common and severe among severe patients upon hospital admission, and AST levels had the strongest correlation with mortality compared to other markers of liver damage. Furthermore, the increase in AST levels was more significant than the increase in ALT levels [21].

In another hospital-based study among 1099 patients with laboratory-confirmed COVID-19, 168/757 (22.2%) had elevated AST, 158/741 (21.3%) had elevated ALT, 76/722 (10.5%) had elevated total bilirubin. Those with elevated liver enzymes had severe COVID-19 disease. The main outcome measures were being admitted to an intensive care unit (ICU), receiving mechanical ventilation, and dying, and several patients with those outcomes were higher, 50% vs. 20.1% in the high AST group, 40.8% vs. 19.9% in high ALT group and 20.8% vs. 9.8% in high total bilirubin group [22].

In another retrospective cohort study conducted in the United States, 2273 patients tested positive out of 3381 patients. For individuals who tested positive, liver injury was categorized based on the level of ALT elevation as none/mild (<2 times the upper limit of normal), moderate (2-5 times the upper limit of normal), and severe (>5 times the upper limit of normal). Individuals who tested positive for COVID had higher median ALT values than those who tested negative, which is evident in both the initial (28 vs. 21 U/L; p<0.001) and peak (45 vs. 25 U/L; P<0.001) values. Furthermore, values greater than two times the upper limit of normal (22% vs. 12%; p<0.001) were more prevalent in positive cases compared to negative cases. However, there was no significant difference in peak ALT values greater than five times the upper limit of normal (6.4% positive vs. 5.0% negative; p=0.12) between the two groups. Patients requiring care in Intensive Care Unit setting were likelier to have a moderate and severe acute liver injury (p<0.001). Among 92 patients who were discharged from the emergency department, 93% had an ALT <2 times the ULN. Out of the 529 patients who required ICU-level care, 54% had an ALT<2 times the ULN, 27% had an ALT 2-5 times the ULN, and 19% had an ALT >5 times the ULN. When all other factors were considered, such as age, body mass index, diabetes, hypertension, intubation, and renal replacement therapy (RRT), the peak ALT level was found to have a significant correlation with death or discharge to hospice (OR, 1.14; P=0.044) in multivariable analysis [15].

In another retrospective cohort study done in 1827 hospitalized patients, liver tests were abnormal in patients with COVID-19 both pre-hospitalization (AST 20.3%, ALT 19.1%, ALP 13.4%, TBIL 4.1%, albumin 27%) and peak hospitalization (AST 83.4%, ALT 61.6%, ALP 22.7%, TBIL 16.1%, albumin 86.6%). The study demonstrated the correlation between abnormal liver tests and severe COVID-19, including the need for ICU admission, mechanical ventilation, and death [23].

Current management strategy

Currently, there is a lack of information on the safety of the medications used to treat COVID-19 individuals with liver injury, and most patients' care is determined by anecdotal experience. This is not different for the population at risk as well. The American Association for the Study of Liver Diseases (AASLD) expert panel's consensus statement [24] has made some recommendations. They recommend that when evaluating patients with COVID-19 and elevated liver biochemical reactions, etiologies unrelated to COVID-19, including other viruses like hepatitis A, hepatitis B, and hepatitis C, should be considered. Consideration should be given to additional factors, such as myositis (particularly glutamic oxalacetic transaminase > ALT), cardiac damage, ischemia, and cytokine release syndrome that might result in higher liver biochemical reactions. Regardless of baseline values, all hospitalized COVID-19 patients should have routine monitoring for biochemical liver markers. This is especially important for those receiving remdesivir or tocilizumab treatment. Severe COVID-19 patients with pre-existing conditions such as advanced liver disease, particularly in elderly patients with concomitant comorbidities, require more intense surveillance or individually tailored therapy methods due to their immunocompromised status. The biochemical markers of liver injury, including ALT/AST, bilirubin, albumin, and prothrombin time, should be examined in all cases of COVID-19 to detect liver damage [24]. For patients with chronic viral hepatitis and COVID-19, antiviral treatment for HBV or HCV infection is not contraindicated. For patients with chronic HBV infection and COVID-19, HBV treatment may be indicated. L-ornithine-L-aspartate administration, a promising treatment for liver failure, may successfully lower ammonia levels in hepatic encephalopathy. Nevertheless, those treatments should not be overemphasized since they are merely complementary approaches. As a result, prebiotics and probiotics may provide a range of health benefits by regulating the microbial balance and bacterial activity in the gastrointestinal tract [25]. Elevated liver biochemistries have been commonly observed in clinical trials of remdesivir, but elevations have rarely been greater than 10 times the baseline values and have rarely led to treatment discontinuation. Circulatory and respiratory mechanical support should be considered for COVID-19 patients with hypoxic hepatitis [26]. For patients with autoimmune hepatitis or liver transplantation, immunosuppressants should be carefully managed based on the severity of the COVID-19 infection. In mild COVID-19 infection, typically, there is no adjustment in chronic immunosuppressant, while in those with new-onset neutropenia or lymphopenia, consideration should be given for careful lowering of their immunomodulators dosage [27].

COVID-19 vaccination

Besides the general management strategies for the treatment of COVID-19 infection, vaccines developed for the prevention of SARS-CoV-2 infection have been considered as the promising approach for fighting against the global pandemic. The efficacy of COVID-19 vaccines in reducing the risk of severe COVID-19-related disease and reduction in COVID-19-associated hospitalizations and deaths has been proven by various studies [28]. The mRNA COVID-19 vaccines Pfizer and Moderna have a vaccine efficacy of 94%-95% compared to a placebo against COVID-19 and are recommended for all adult patients with Chronic Liver Disease and organ transplant recipients [29]. CDC has recommended people who are pregnant and people who are breastfeeding remain up-to-date with COVID-19 vaccination. CDC has also recommended vaccines for children and adolescents aged six months and older.

## Conclusions

The potential for liver injury in patients with COVID-19 infection must be taken into consideration. Extra vigilance is required in patients in the high risks group and age. Mild liver injury generally tends to recover with conservative management. Patients on treatment for chronic infectious liver conditions, autoimmune hepatitis and liver transplant recipients should not have their treatment abruptly stopped. Based on the contemporary data, acute liver failure due to COVID-19 per se is not very common even in high-risk and certain age group patients except in those with shock and multiorgan dysfunction.
